# The complete mitochondrial genome of *Plagiodera versicolora* (Laicharting)(Coleoptera: Chrysomelidae)

**DOI:** 10.1080/23802359.2020.1829138

**Published:** 2020-10-21

**Authors:** Wei-Wei Xie, Liang-Jing Sheng, Yi Wan, Xiao-Qian Weng, Guang-Hong Liang, Fei-Ping Zhang, Hui Chen

**Affiliations:** a College of Forestry, Fujian Agriculture and Forestry University, Fuzhou, China; b Key Laboratory of Integrated Pest Management in Ecological Forests, Fujian Province University, Fujian Agriculture and Forestry University, Fuzhou, China

**Keywords:** Complete mitochondrial genome, *Plagiodera versicolora*, phylogenomic analysis

## Abstract

*Plagiodera versicolora* (Laicharting) is a leaf-eating pest widely distributed in the world. In this study, the first complete mitochondrial genome of *P. Versicolora* (Laicharting) was assembled and analyzed. The complete mitochondrial genome of *P. Versicolora* (Laicharting) is 16,857 bp with 22.39% GC containing, 13 protein-coding genes, 22 transfer RNA (tRNA), 2 ribosomal RNA (rRNA), as well as an AT-rich region. Phylogenomic analysis indicated that *P. Versicolora* (Laicharting) is sister to *Chrysomela populi*This study provides useful information for the identification of this species and the study of genetic evolution with other species of Chrysomelidae.

*Plagiodern Versicolora* (Laicharting) is classified as Chrysomelida of Coleoptera, which is one of the major pests of poplar and willow (Wade 1994).This insect is widely distributed in the world, including Asia, Europe and North America. However, its genome information is still unclear at present (De et al. 2005). In this study, the complete mitochondrial genome of *P. Versicolora* (Laicharting) was reported for the first time, which is of great significance for understanding its evolution and population genetics.

The samples of *P. Versicolora* (Laicharting) were collected by the traps from Minhou, Fujian Province, China (119°4′35″E, 26°14′9″N). All voucher specimens were assigned with a unique code and deposited in the Key Laboratory of Integrated Pest Management in Ecological Forests, Fujian Province University, Fujian Agriculture and Forestry University (voucher no.YJ-202007). Total genomic DNA were extracted from the legs of samples using TruSeq DNA sample Preparation kit (Vanzyme, China). DNA quality and concentration were determined using Nanodrop (Thermo Fisher Scientific, Waltham, MA, USA). The multiple samples were mixed and sequenced by Illumina Hiseq 2500 (Genesky Biotechnologies Inc. Shanghai, China).

A total of 51,021,324 clean reads were obtained from the 53,740,482 raw reads after filtration. After the de novo assembly of metaSPAdes (Nurk et al. 2017), a 16,857 bp length complete mitochondrial genome of *P. Versicolora* (Laicharting) with 22.39% GC content was obtained (GenBank accession No: MT826862). Three characteristics of protein-coding sequence, tRNA, and rRNA were obtained by mitoMaker (Bernt et al. 2013). Also, tRNA genes were predicted by tRNAscan software (Lowe and Eddy 1997). Finally, a total of 37 genes were annotated, including 13 protein-coding genes, 22 transfer RNA (tRNA), and 2 ribosomal RNA(rRNA), as well as and an AT-rich region. To confirm the phylogeny of *P. Versicolora* (Laicharting), the other 11 complete genomes were obtained from GenBank and were aligned using HomBlocks software (Bi et al. 2018). A neighbor-joining (NJ) tree (Saitou 1987) with 1000 bootstrap replicates was performed by MEGA 7.0 (Sudhir et al. 2016). The result of NJ phylogenetic tree indicate that *P. Versicolora* (Laicharting) is closely related to *Chrysomela populi* (Figure 1). The mitochondrial genome of *P. Versicolora* (Laicharting) will provide useful genetic information for further study on genetic diversity and genetic evolution of Chrysomelidae species.

**Figure 1.**
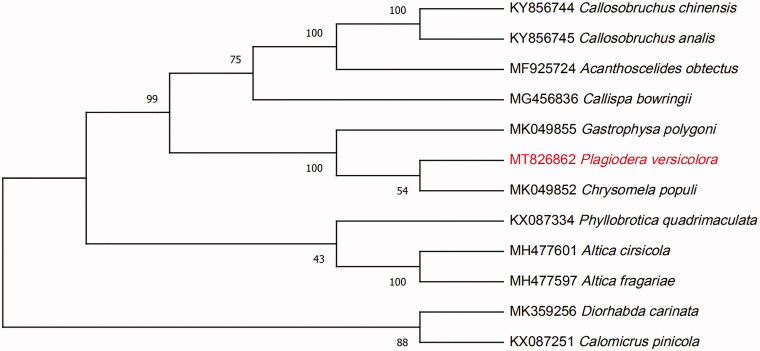
The neighbor-joining (NJ) phylogenetic tree based on 12 mitochondrial genome. Values along branches correspond to ML bootstrap percentages.

## Data Availability

The data that support the findings of this study are openly available in “NCBI” at https://www.ncbi.nlm.nih.gov/, reference number MT826862.
